# Predictors of mental illness onset in adolescents and adults with intellectual disability: A retrospective cohort study in New South Wales, Australia

**DOI:** 10.1177/00048674251374483

**Published:** 2025-09-28

**Authors:** Stefan C Michalski, Yunhe Huang, Preeyaporn Srasuebkul, Rachael C Cvejic, Samuel RC Arnold, Julian N Trollor

**Affiliations:** 1 National Centre of Excellence in Intellectual Disability Health, Faculty of Medicine and Health, UNSW Sydney, Sydney, NSW, Australia; 2 Department of Paediatrics, Faculty of Medicine, Dentistry and Health Sciences, University of Melbourne, Parkville, VIC, Australia; 3 School of Psychology, Western Sydney University, Penrith, NSW, Australia

**Keywords:** Intellectual disability, mental illness, serious mental illness, risk factors, survival analysis, comorbidities

## Abstract

**Background:**

People with intellectual disability are disproportionately affected by mental illness, including serious mental illness. While the prevalence of mental illness in this population is well-documented, the factors associated with the onset of any mental illness and serious mental illness lack comprehensive investigation. This study aims to identify demographic, service-related and disability-related factors associated with the onset of any mental illness and serious mental illness in people with intellectual disability using a large, linked dataset in New South Wales, Australia.

**Methods:**

A retrospective cohort study was conducted using linked administrative data for 47,330 individuals with intellectual disability aged 13–80 years. Data from 2004 to 2018 were used to track first recorded contact with mental health services for any mental illness and serious mental illness. Flexible parametric survival analysis was employed to account for time-varying factors and estimate hazard ratios for the risk of developing any mental illness or serious mental illness.

**Results:**

Nearly half of the cohort (48.9%) experienced any mental illness, and 11.7% experienced serious mental illness. Factors associated with any mental illness included attention-deficit/hyperactivity disorder, learning disorders, physical comorbidities, and living in areas of greater socioeconomic disadvantage. Serious mental illness onset was associated with living in outer regional, remote or very remote areas, attention-deficit/hyperactivity disorder, learning disorders, male sex, and a history of any mental illness.

**Conclusion:**

This study identified factors associated with the onset of any mental illness and serious mental illness in people with intellectual disability. These findings emphasise the need for early identification and targeted interventions to improve mental health outcomes in this high-risk population.

## Background

People with intellectual disability experience significantly higher rates of mental illness compared to the general population (Hughes-McCormack et al., 2017; McMahon and Hatton, 2021; Perera et al., 2020; Sheehan et al., 2015). In Australia, people with intellectual disability comprise approximately 1% of the population but account for 6.3% of individuals accessing publicly funded mental health services, and 12% of total public mental health expenditures (Srasuebkul et al., 2021). A recent study found that the prevalence of mental disorders in people with intellectual disability in New South Wales (NSW) is nearly double that of the general population, with the prevalence of serious mental illness (SMI) to be more than triple (Arnold et al., 2025). People with intellectual disability are overrepresented within mental health services (Srasuebkul et al., 2021), which is driven by a combination of systemic barriers (Whittle et al., 2018) and unmet needs (Nicholson et al., 2022; Salvador-Carulla and Symonds, 2016). Despite disproportionate representation of mental illness, the factors contributing to the elevated risk and onset of mental illness in people with intellectual disability remain underexplored.

The mental health of people with intellectual disability is strongly associated with a combination of social determinants, environmental factors and health-related factors (Totsika et al., 2022). Discrimination, stigma, social exclusion, lower income, under-employment and housing insecurity, and poor living conditions are significant contributors to poor mental health outcomes (Ali et al., 2015; Emerson et al., 2011; Martínez-Cao et al., 2021; Whitney et al., 2019). Furthermore, personal and health-related factors, including traumatic life events, sex, inadequate support, lower cognitive ability and chronic health conditions like diabetes or poor sleep have been found to exacerbate mental health risks (Bond et al., 2020; Cooper et al., 2007; de Winter et al., 2015; Harper et al., 2023; Scott and Havercamp, 2014; Wigham and Emerson, 2015). These factors are interconnected and contribute to mental health outcomes for people with intellectual disability.

Despite extensive research documenting factors associated with poor mental health in people with intellectual disability, there remains a critical gap in longitudinal evidence identifying factors associated with the onset of mental illness in this population. The existing evidence base is largely derived from clinical samples and cross-sectional studies, limiting both the ability to establish causal relationships and the generalisability of findings to broader populations with intellectual disability (Buckley et al., 2020; Totsika et al., 2022). A recent meta-analysis found that few studies explored demographic or clinical factors associated with psychiatric comorbidity in people with intellectual disability, and these data were too inconsistent to support meaningful conclusions (Mazza et al., 2020). Large-scale longitudinal studies with comprehensive population coverage are needed to identify factors associated with mental illness onset in people with intellectual disability.

This study aims to identify the demographic, health service-related and disability-related factors associated with the development of any mental illness and SMI in adolescents and adults with intellectual disability. To our knowledge, this is the first study in Australia to determine the factors associated with any mental illness onset and SMI onset in a large cohort of people with intellectual disability. Identifying these underlying factors is crucial for improving mental health policy, services, outcomes and quality of life for people with intellectual disability.

## Methods

### Datasets and record linkage

This study uses an existing large linked administrative dataset of people with intellectual disability in NSW, Australia, described elsewhere (Reppermund et al., 2024). The cohort of people with intellectual disability includes all people with a recorded diagnosis of intellectual disability across multiple health and disability service datasets. The NSW Centre for Health Record Linkage (CHeReL) and the Australian Institute of Health and Welfare (AIHW) performed linkage and de-identification. See Supplementary Material S1 for a description of the datasets used to identify variables in this study.

### Study population

This study includes all people with intellectual disability who were alive and aged 13–80 years during the study period of 1 January 2004–31 December 2018, and had no recorded mental illness at start of follow-up (1 January 2004 or their 13th birthday, whichever was latest). We selected this age range because adolescence and adulthood are important life stages for mental illness onset (de Girolamo et al., 2012). Following previous work, we used a 2.5-year lookback period from 1 July 2001–31 December 2003 to exclude individuals with pre-existing mental illness (Li et al., 2018). Follow-up for each individual ended on the date of first outcome (calculated separately for analyses involving each outcome), 31 December 2018, date of death, or their 81st birthday, whichever was earliest.

### Outcomes

For both outcomes, we used the first date of record as the date of onset. We defined any mental illness as one or more of the following: (1) diagnosis of a non-organic and non-neurodevelopmental psychiatric disorder in the Admitted Patient Data Collection (APDC), Mental Health Ambulatory Data Collection (MHAMB) or Emergency Department Data Collection (EDDC) datasets, based on International Classification of Diseases and Related Health Problems, 10th Revision, Australian Modification (ICD-10-AM) diagnostic codes F10-F63, F65-F69, F91-F94, F98.0-F98.3, F99, R45.81, X60-X84, Y87.0, U79.1-U79.3 or equivalent; (2) admission to an inpatient psychiatric unit in the APDC; or (3) record of mental health service use from the MHAMB or Medicare Benefits Schedule (MBS) datasets. See Supplementary material S2 for details of outcome ascertainment.

We defined SMI as one or more of the following: (1) any hospital admission where the principal diagnosis is a non-organic and non-neurodevelopmental psychiatric disorder in the APDC, as described above; (2) admission to an inpatient psychiatric unit in the APDC; or (3) any of the following ICD-10-AM diagnoses in the MHAMB: F20, F25, F23, F30, F31, F32.3, F33.3. We did not use the EDDC for SMI ascertainment due to limited diagnostic certainty in emergency settings (Woo et al., 2006).

### Study variables

We included the following variables in this study: sex, remoteness of residence area (Australian Bureau of Statistics, 2018a), area-based index of relative socioeconomic disadvantage (IRSD; Australian Bureau of Statistics, 2018b), specific neuropsychiatric conditions (autism, attention-deficit/hyperactivity disorder [ADHD] and learning disorders, cerebral palsy, epilepsy, Down syndrome, other congenital syndromes) and physical comorbidity. Remoteness categories are based on the Australian Bureau of Statistics Australian Statistical Geography Standard, which classifies areas according to relative access to services measured by road distance to service centres (Australian Bureau of Statistics, 2023). We treated missing remoteness area/IRSD as a separate category in analyses. We defined physical comorbidity as an ordinal variable (0, 1, 2, 3 or more conditions) representing the number of co-occurring chronic physical health conditions from a pre-defined list of ICD-10 codes, ascertained using APDC data (Huang et al., 2025). We treated epilepsy and physical comorbidity as time-varying variables. We recorded an individual as having epilepsy from 1 January of the year after first diagnosis to the end of follow-up. For physical comorbidity, we applied any changes to comorbidity level (i.e. diagnosis of first, second and third eligible conditions) from 1 January of the following year to the end of follow-up. Details on definitions of neuropsychiatric conditions and physical comorbidities are in Supplementary material S2.

### Statistical analysis

We used Stata 18 for all analyses (StataCorp, 2023). We conducted flexible parametric survival analysis using Stata package *stpm3* (Lambert, 2023) to examine risk factors for each outcome because most variables did not meet the proportional hazards assumption. We split the data by each calendar year in the study period to account for time-varying variables, using age (in years) at 1 January of each calendar year as the time scale. This approach ensures that all hazard ratios are age-adjusted, as the survival models account for the underlying age-related risk of mental illness onset. We adjusted for Aboriginal/Torres Strait Islander status as a covariate but do not present results related to this variable, in keeping with the limits of our ethical clearance.

Along with unadjusted models for each variable, we analysed separate individually adjusted models for sets of variables according to their causal relationship, where each model only included the variable of interest and its confounders (see Supplementary material 3). This approach allows us to estimate each variable’s total effect (direct effect + effect of mediators in the causal chain) on the outcome (Westreich and Greenland, 2013). As a secondary analysis to explore variations in hazard ratio over time, we used Stata package *standsurv* to estimate the predicted values of standardised hazard ratios at each year of age (Lambert, 2021).

## Results

**Figure 1.**
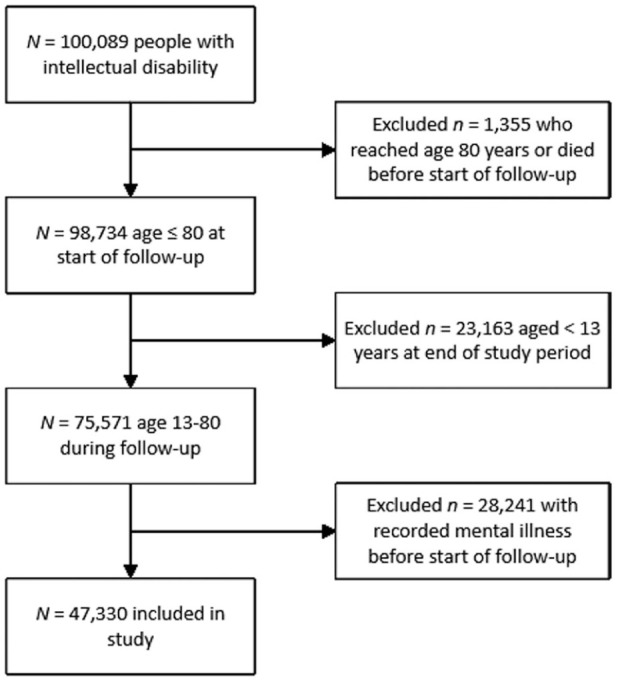
Cohort selection.

This study included 47,330 eligible individuals from the original cohort (Figure 1). Total length of follow-up was 488,374.15 years (M = 10.32, SD = 4.94). Our cohort was predominantly male and mainly lived in major cities and socioeconomically disadvantaged areas (Table 1).

**Table 1.** Cohort characteristics (*N* = 47,330).

	*n* (%)
Age at start of follow-up (M, SD)	23.36 (15.58)
Sex	
Male	28,802 (60.9%)
Female	18,528 (39.1%)
Remoteness area	
Major cities	26,311 (55.6%)
Inner regional	11,087 (23.4%)
Outer regional	3855 (8.1%)
Remote	215 (0.5%)
Very remote	217 (0.5%)
Missing	5645 (11.9%)
Index of relative socioeconomic disadvantage	
First quintile (most disadvantaged)	12,256 (25.9%)
Second quintile	10,762 (22.7%)
Third quintile	9698 (20.5%)
Fourth quintile	6801 (14.4%)
Fifth quintile (least disadvantaged)	5302 (11.2%)
Missing	2511 (5.3%)
Neuropsychiatric conditions^a^	
Autism and related conditions	12,085 (25.5%)
ADHD and learning disorders	5586 (11.8%)
Cerebral palsy	3578 (7.6%)
Epilepsy	7362 (15.6%)
Down syndrome	3012 (6.4%)
Other congenital syndromes	462 (1.0%)
Physical comorbidity^a^	
None	19,685 (41.6%)
One condition	7770 (16.4%)
Two conditions	4780 (10.1%)
Three or more conditions	15,095 (31.9%)

a Includes all individuals with these diagnoses recorded during the lookback or study period.

### Factors associated with any mental illness

**Figure 2.**
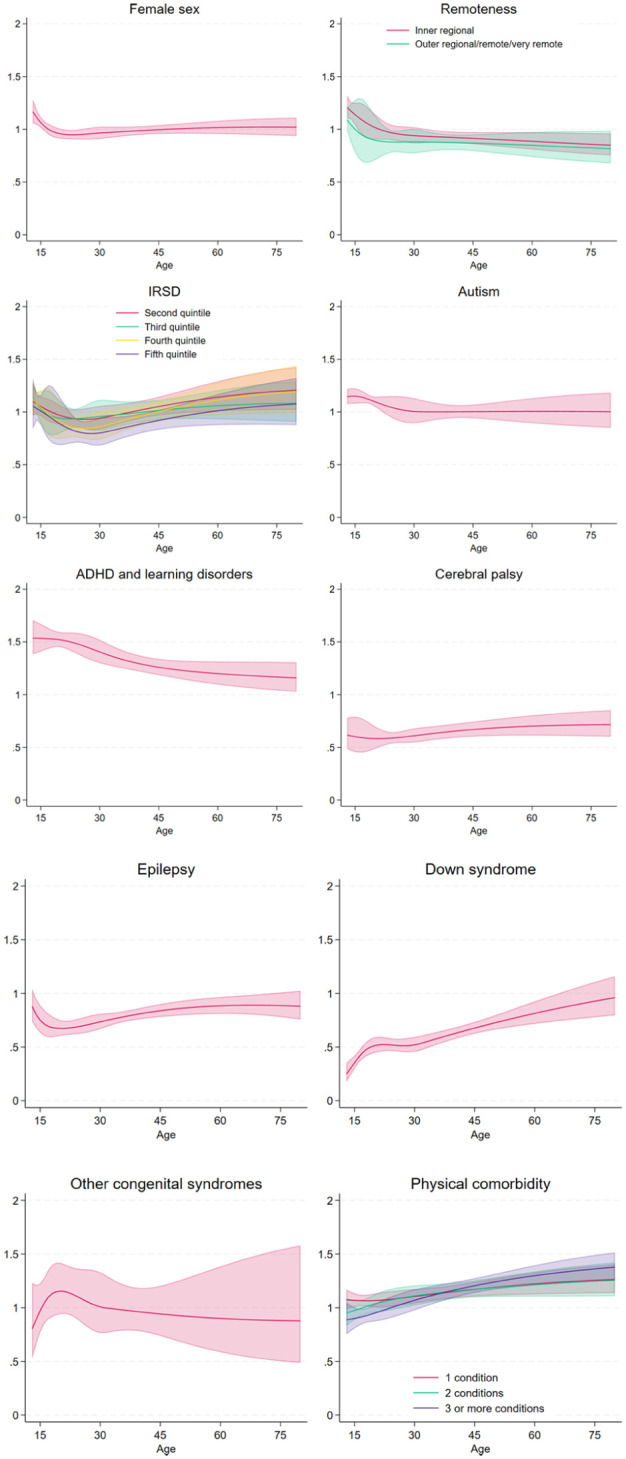
Standardised hazard ratios for the onset of any mental illness by age and risk factor.

Nearly half of our cohort (*n* = 23,121, 48.9%) experienced any mental illness during follow-up. Table 2 shows outputs of models examining the onset of any mental illness. Individually adjusted models show that living in an area with average or greater disadvantage, diagnosis of ADHD or learning disorders, and having one or more physical comorbidities was significantly associated with increased risk of any mental illness onset. Conversely, having cerebral palsy or Down syndrome was associated with decreased risk. Figure 2 presents the standardised hazard ratios for the onset of any mental illness by age for each factor.

**Table 2.** Factors associated with onset of any mental illness.

	Unadjusted		Individually adjusted	
Variable	Hazard ratio (95% CI)	*p*	Hazard ratio (95% CI)	*p*
Female sex	1.05 (0.99, 1.11)	0.077	1.05 (0.99, 1.11)	0.077
Remoteness area^a^ (ref: Major cities)				
Inner regional	1.03 (0.97, 1.09)	0.368	1.00 (0.94, 1.06)	0.923
Outer regional/remote/very remote	1.14 (0.74, 1.77)	0.553	0.95 (0.86, 1.05)	0.307
Area-based index of socioeconomic disadvantage (ref: Most Disadvantaged)^a^				
More Disadvantaged	1.07 (1.00, 1.13)	0.043	1.09 (1.02, 1.17)	0.013
Average	1.05 (0.98, 1.13)	0.185	1.09 (1.02, 1.17)	0.013
Less Disadvantaged	1.07 (0.96, 1.19)	0.222	1.06 (0.97, 1.15)	0.175
Least Disadvantaged	0.97 (0.88, 1.08)	0.606	0.94 (0.83, 1.08)	0.397
Neuropsychiatric conditions				
Autism	1.07 (1.01, 1.14)	0.024	1.04 (0.98, 1.10)	0.171
ADHD and learning disorders	1.36 (1.27, 1.45)	0.000	1.39 (1.32, 1.46)	0.000
Cerebral palsy	0.59 (0.55, 0.65)	0.000	0.64 (0.59, 0.69)	0.000
Epilepsy	0.97 (0.70, 1.34)	0.844	1.06 (0.51, 2.21)	0.880
Down syndrome	0.61 (0.56, 0.66)	0.000	0.61 (0.56, 0.66)	0.000
Other congenital syndromes	0.93 (0.76, 1.14)	0.504	0.91 (0.75, 1.12)	0.393
Physical comorbidity (ref: none)				
1 condition	1.10 (1.01, 1.20)	0.035	1.14 (1.08, 1.20)	0.000
2 conditions	2.13 (0.79, 5.71)	0.135	1.09 (1.03, 1.16)	0.006
3 or more conditions	0.96 (0.87, 1.06)	0.406	1.11 (1.06, 1.17)	0.000

a Combined outer regional, remote, and very remote due to small numbers, omitted hazard ratios for missing remoteness/area-based index of socioeconomic disadvantage due to lack of interpretability.

### Factors associated with SMI

**Figure 3.**
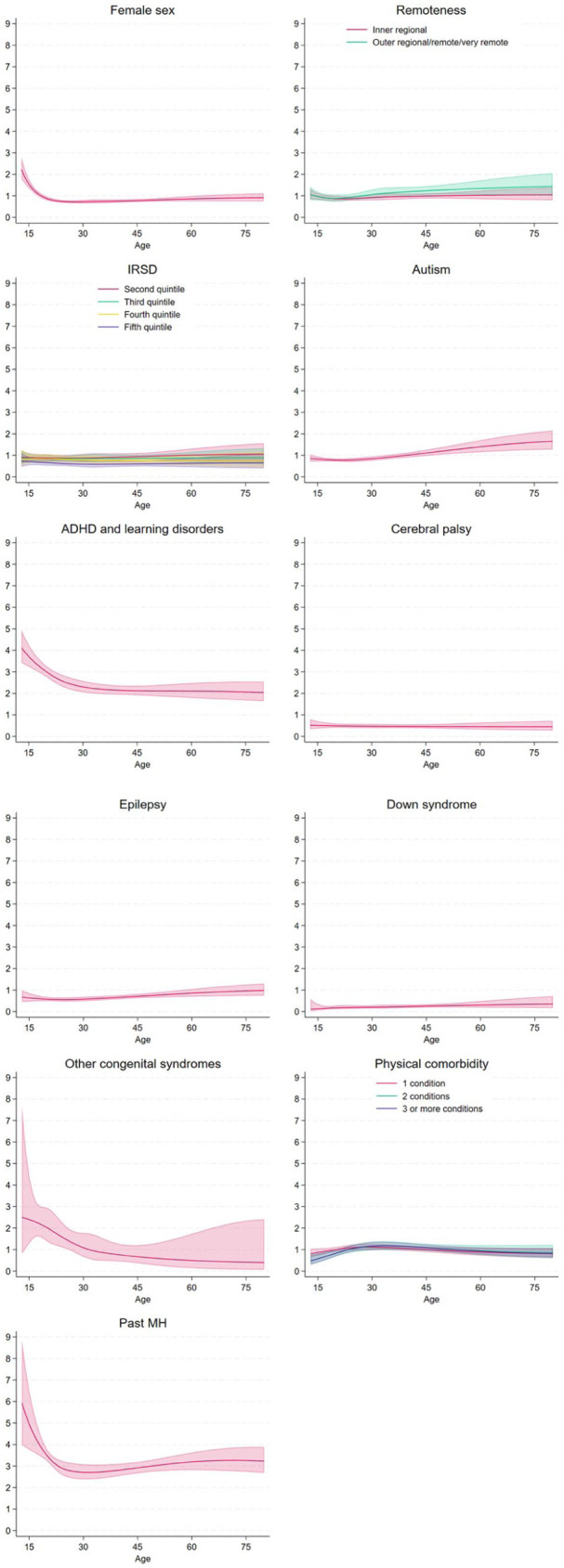
Standardised hazard ratios for the onset of serious mental illness by age and risk factor.

Approximately 11.7% of our cohort (*n* = 5554) experienced SMI during follow-up. Table 3 shows outputs of models examining SMI onset. Individually adjusted models show that living in outer regional, remote or very remote areas, living in a more disadvantaged area, diagnosis of ADHD or learning disorders, and history of any mental illness were associated with increased risk of SMI onset, while being female, living in the least disadvantaged area, and having Down syndrome was associated with decreased risk. Figure 3 presents the standardised hazard ratios for the onset of SMI by age for each factor.

**Table 3.** Factors associated with serious mental illness onset.

	Unadjusted		Individually adjusted	
Variable	Hazard ratio (95% CI)	*p*	Hazard ratio (95% CI)	*p*
Female sex	0.87 (0.81, 0.93)	0.000	0.87 (0.81, 0.93)	0.000
Remoteness area (ref: major cities)				
Inner regional	1.10 (1.01, 1.20)	0.027	0.97 (0.88, 1.06)	0.459
Outer regional/remote/very remote	1.49 (1.33, 1.67)	0.000	1.20 (1.06, 1.37)	0.005
Area-based index of socioeconomic disadvantage (ref: Most Disadvantaged)				
More Disadvantaged	1.03 (0.93, 1.14)	0.572	1.58 (1.44, 1.73)	0.000
Average	0.89 (0.80, 0.98)	0.024	1.05 (0.94, 1.16)	0.388
Less Disadvantaged	0.76 (0.67, 0.85)	0.000	0.93 (0.84, 1.04)	0.202
Least Disadvantaged	0.59 (0.52, 0.68)	0.000	0.82 (0.72, 0.92)	0.001
Neuropsychiatric conditions				
Autism	1.18 (1.04, 1.34)	0.010	1.09 (0.96, 1.23)	0.189
ADHD and learning disorders	2.44 (2.23, 2.68)	0.000	2.52 (2.30, 2.76)	0.000
Cerebral palsy	0.46 (0.38, 0.55)	0.000	0.45 (0.38, 0.54)	0.000
Epilepsy	0.73 (0.64, 0.83)	0.000	0.70 (0.61, 0.79)	0.000
Down syndrome	0.25 (0.19, 0.32)	0.000	0.25 (0.19, 0.32)	0.000
Other congenital syndromes	1.00 (0.72, 1.39)	0.986	1.01 (0.72, 1.42)	0.947
Physical comorbidity (ref: none)				
1 condition	0.87 (0.79, 0.97)	0.009	0.97 (0.88, 1.08)	0.618
2 conditions	0.83 (0.73, 0.94)	0.004	0.98 (0.87, 1.12)	0.801
3 or more conditions	0.80 (0.73, 0.88)	0.000	0.97 (0.88, 1.08)	0.611
History of any mental illness	6.38 (4.46, 9.12)	0.000	3.69 (3.32, 4.11)	0.000

## Discussion

This study identified factors associated with the onset of any mental illness and SMI in adolescents and adults with intellectual disability. In this study, data from 47,330 individuals with intellectual disability revealed that nearly half (48.9%) experienced onset of mental illness, and 11.7% experienced onset of SMI. Several factors increased the risk of onset for any mental illness, including socioeconomic disadvantage, ADHD and learning disorders and the presence of physical comorbidities. Factors that increased the onset of SMI included living in outer regional/remote/very remote areas, socioeconomic disadvantage, ADHD and learning disorders, male sex and a history of any mental illness.

### Key findings

Our current study showed that female sex was not significantly associated with onset of any mental illness and was associated with lower risk of SMI onset. This contrasts with our previous descriptive study using the same cohort, which showed that females had slightly lower prevalence of any mental illness, and slightly higher prevalence of SMI, than males (Arnold et al., 2025). This discrepancy is likely due to the nature of survival analysis, which accounts for the timing of onset in addition to the overall likelihood of mental illness. Sex differences in help-seeking behaviour, access to healthcare and diagnostic practices may also contribute to differences in recorded SMI (Axmon et al., 2017; Whittle et al., 2024). In addition, diagnoses such as substance use and behaviour disorders, which are more prevalent among males (Axmon et al., 2017; Lunsky et al., 2009), are more likely to result in psychiatric hospitalisation or outpatient service use, and thus may increase the likelihood of meeting our inclusion criteria of SMI (Morgan et al., 2014). Future longitudinal research would help provide greater clarity into the relationship between sex and mental illness onset in this population.

Patterns of geographic and socioeconomic disparities varied by outcome and may have been shaped by contextual factors such as healthcare access. Greater geographical remoteness was associated with slightly increased risk of SMI onset but not any mental illness, and higher socioeconomic disadvantage was associated with greater risks of both outcomes. However, the most disadvantaged category did not always show the highest mental illness risks. As our study uses healthcare records to capture outcomes, barriers to healthcare associated with remoteness and socioeconomic disadvantage are likely to have impacted these findings. Poorer access to healthcare services in remote and disadvantaged areas can hinder prevention and early identification (thus capturing fewer outcome events), contributing to more severe presentations by the time services are accessed (Burton and Walters, 2013; Iacono et al., 2003; Kavanagh et al., 2023; Shea et al., 2022; Wark et al., 2015). Given this disparity, expanding cost-effective service models, such as telehealth and stepped-care approaches, could improve access for disadvantaged groups by reducing out-of-pocket costs and logistical barriers (Selick et al., 2021; Szeftel et al., 2012). Our findings reveal significant associations between neuropsychiatric conditions and the risk of developing any mental illness and SMI. Notably, ADHD and learning disorders were strongly associated with both outcomes. This is consistent with extensive literature identifying high rates of comorbidity between ADHD and various mental health conditions in the general population (Choi et al., 2022; Kessler et al., 2006; Larson et al., 2011), as well as elevated risks of severe psychiatric outcomes such as self-injury and suicide (Hinshaw et al., 2012; Miklowitz, 2015). Emerging research indicates that ADHD is more prevalent and frequently undiagnosed in people with intellectual disability (Al-Khudairi et al., 2019; Baker et al., 2010; Perera et al., 2022). There is a need for targeted interventions that support accurate diagnosis and address both ADHD symptoms and associated psychiatric comorbidities (Larson et al., 2011; Miller et al., 2020; Perera et al., 2022; Sun et al., 2019).

Epilepsy was associated with reduced risk of SMI onset but not any mental illness. Past research suggests a complex relationship between epilepsy and psychiatric disorders in people with intellectual disability. A review by Brizard et al. (2021) found no significant relationship between epilepsy and psychiatric disorder rates in adults with intellectual disability. This contrasts significantly with the general population, where meta-analyses have consistently identified higher rates of depression, anxiety, and psychosis in adults with epilepsy (Buranee et al., 2016; Clancy et al., 2014; Fiest et al., 2013; Hellwig et al., 2012; Scott et al., 2017). Psychiatric care for individuals with epilepsy may be delivered within neurology or neuropsychiatry services, rather than mainstream mental health services, leading to underrepresentation in administrative datasets used to define SMI. The potential mood-stabilising effects of anti-epileptic medications may also contribute to lowered SMI rates.

Furthermore, our study found that having Down syndrome and/or cerebral palsy was associated with decreased risk of both any mental illness and SMI onset. Our finding regarding Down syndrome is consistent with our previous prevalence study using the same linked dataset (Arnold et al., 2025). There is evidence suggesting that Down syndrome may confer resilience or offer protective factors to mental illness in comparison with other people with intellectual disability (Mantry et al., 2008). However, there is considerable evidence suggesting the prevalence of mental illness among people with cerebral palsy is high, with Downs et al. (2018) finding that people with cerebral palsy who also have intellectual disability are at high risk of mental health symptoms. For people with cerebral palsy, psychiatric care may be more frequently embedded within multidisciplinary rehabilitation settings rather than mainstream mental health services (Trabacca et al., 2016). Research also suggested that motor and communication impairments in cerebral palsy may interfere with accurate identification of mental illness (Bhatnagar et al., 2024). These could contribute to the lower recorded rates of mental illness and SMI in our dataset despite the presence of underlying psychiatric symptoms.

Finally, no significant associations were found between autism with the onset of any mental illness and SMI. These findings are consistent with our previous prevalence study (Arnold et al., 2025), but contrasts with existing literature reporting elevated rates of mood, anxiety and psychotic disorders in people with intellectual disability on the autism spectrum (Bakken et al., 2010; Cervantes and Matson, 2015; Lai et al., 2019; Lugo-Marin et al., 2019; Peña-Salazar et al., 2022). This discrepancy may be due to our inability to capture undiagnosed and/or unrecorded cases of autism and mental illness using administrative datasets. Specifically, the known under-identification of autism in older age groups (Hwang et al., 2019) is a major barrier to accurate estimation of mental illness risks in this population.

In this study, we found that the presence of one or more physical comorbidities significantly increased the risk of developing any mental illness but not SMI onset. This finding is consistent with previous research showing that physical health issues can exacerbate the risk of mental health disorders (Launders et al., 2022a, 2022b; Onyeka et al., 2019; Tegethoff et al., 2016). However, the lack of association with SMI may be because conditions like psychosis, which make up a large part of SMI, typically emerge earlier in life, before physical health issues have a significant impact. The younger age of our sample may partly account for this finding, as individuals in this age group may not yet have developed the chronic physical conditions that typically appear later in life (Stubbs et al., 2016). Another possibility is that individuals with SMI are less likely to have physical comorbidities diagnosed at onset due to the compartmentalisation of mental and physical healthcare, which can result in underdiagnosis or insufficient documentation (Happell et al., 2012). Alternatively, integrated care models often implemented for people with SMI (Rodgers et al., 2018), particularly for people with psychotic conditions, may better support their physical health needs, potentially mitigating the impact of physical comorbidities on SMI risk.

### Implications

This study extends knowledge about factors associated with mental illness and SMI among people with intellectual disability and provides insights into the complexity and associations of mental illness within this population. These findings add to the growing body of evidence supporting the urgent need for targeted action in Australia. Incremental progress has been made, such as the establishment of tertiary Intellectual Disability Mental Health (IDMH) services in NSW and new mental health teams within mainstream public mental health services in Queensland. Notably, the IDMH service hubs in NSW provide in-person and telehealth assessment and care planning in addition to professional training, which have significantly improved service access and outcomes (O’Shea et al., 2023). Future use of updated linked data could help evaluate its impact. However, overall service provision in most jurisdictions of Australia remains patchy, inadequate or non-existent, and there is a projected shortage of psychiatrists with expertise in this area (Cvejic et al., 2018). The Disability Royal Commission (DRC) recommendation 6.33 called for the establishment of multidisciplinary health teams within local health services and statewide networks (Royal Commission into Violence Abuse Neglect and Exploitation of People with Disability, 2020). As per a previous national consensus statement (Department of Developmental Disability Neuropsychiatry, UNSW Sydney, 2018), these recommendations should also be applied to mental health services to ensure comprehensive, multidisciplinary support that effectively addresses the significant mental health needs of this population. However, except for Queensland, and the aforementioned tertiary service in NSW, jurisdictions have offered a muted response to recommendations to better equip mainstream mental health services. We continue to strongly advocate for the development of such services to optimise inefficient care pathways and address the ‘revolving door’ phenomenon experienced by people with intellectual disability and mental illness, which leads to high rates of hospital presentations and inefficient care delivery (Cvejic et al., 2022; Li et al., 2018; Srasuebkul et al., 2021).

### Limitations

Several methodological considerations and data limitations must be acknowledged. The recorded onset of mental illness in administrative datasets is likely to occur later than the actual onset, as these data reflect service use rather than initial symptom emergence. The lack of full diagnostic information in datasets such as the EDDC and MBS may have masked the identification of co-occurring SMI. Furthermore, we do not have records of NSW residents who do not access services or use hospitals or emergency departments in other states in Australia. Although our dataset captures a significant proportion of people with intellectual disability in NSW, people with mild intellectual disability may be under-represented as we only capture people who have been recognised by the healthcare or disability service systems. Finally, data linkage is subject to potential clerical errors. The potential for false positives in data linkage is minimal as CHeReL aims for a false positive rate of 0.5%.

### Conclusion

This study identified key factors associated with the onset of mental illness and SMI among people with intellectual disability, including sex, remoteness, socioeconomic disadvantage, neuropsychiatric conditions and physical comorbidities. Our results contribute to a growing body of evidence advocating for urgent action to enhance mental health services for people with intellectual disability in Australia. Current service provision remains inadequate, and there is a critical need for multidisciplinary health teams, telehealth expansion and policy reforms to ensure equitable and effective care. Addressing these gaps is essential to reducing the disproportionate burden of mental illness and improving health outcomes in this population.

## Supplemental Material

sj-docx-1-anp-10.1177_00048674251374483 – Supplemental material for Predictors of mental illness onset in adolescents and adults with intellectual disability: A retrospective cohort study in New South Wales, Australia Supplemental material, sj-docx-1-anp-10.1177_00048674251374483 for Predictors of mental illness onset in adolescents and adults with intellectual disability: A retrospective cohort study in New South Wales, Australia by Stefan C Michalski, Yunhe Huang, Preeyaporn Srasuebkul, Rachael C Cvejic, Samuel RC Arnold and Julian N Trollor in Australian & New Zealand Journal of Psychiatry

sj-docx-2-anp-10.1177_00048674251374483 – Supplemental material for Predictors of mental illness onset in adolescents and adults with intellectual disability: A retrospective cohort study in New South Wales, Australia Supplemental material, sj-docx-2-anp-10.1177_00048674251374483 for Predictors of mental illness onset in adolescents and adults with intellectual disability: A retrospective cohort study in New South Wales, Australia by Stefan C Michalski, Yunhe Huang, Preeyaporn Srasuebkul, Rachael C Cvejic, Samuel RC Arnold and Julian N Trollor in Australian & New Zealand Journal of Psychiatry

sj-docx-3-anp-10.1177_00048674251374483 – Supplemental material for Predictors of mental illness onset in adolescents and adults with intellectual disability: A retrospective cohort study in New South Wales, Australia Supplemental material, sj-docx-3-anp-10.1177_00048674251374483 for Predictors of mental illness onset in adolescents and adults with intellectual disability: A retrospective cohort study in New South Wales, Australia by Stefan C Michalski, Yunhe Huang, Preeyaporn Srasuebkul, Rachael C Cvejic, Samuel RC Arnold and Julian N Trollor in Australian & New Zealand Journal of Psychiatry
