# Supplementary material for: Predictors of mental illness onset in adolescents and adults with intellectual disability: A retrospective cohort study in New South Wales, Australia
Source: Aust N Z J Psychiatry. 2025 Sep 28;59(12):1095–105. doi: 10.1177/00048674251374483 (PMC12634903; doi:10.1177/00048674251374483)
Supplement: sj-docx-1-anp-10.1177_00048674251374483 – Supplemental material for Predictors of mental illness onset in adolescents and adults with intellectual disability: A retrospective cohort study in New South Wales, Australia [file sj-docx-1-anp-10.1177_00048674251374483.docx]

**Supplementary Table 1**

*Datasets used for variable ascertainment*

| Name | Dates available | Description | Diagnostic code set | Variables |
| --- | --- | --- | --- | --- |
| Admitted Patient Data Collection (APDC) | 1 Jul 2001-30 Jun 2021 | All admissions to NSW public and private hospitals, including admission/discharge dates and up to 51 diagnoses | ICD-10-AM | Mental ill-health, serious mental illness, neurodevelopmental comorbidities, physical comorbidity |
| Mental Health Ambulatory Data Collection (MH-AMB) | 1 Jan 2001-30 Jun 2021 | Diagnosis and service-related information for all visits to NSW public community mental health services | ICD-10-AM | Mental ill-health, serious mental illness, neurodevelopmental comorbidities |
| Emergency Department Data Collection (EDDC) | 1 Jan 2005-30 Jun 2021 | Date, reason, and separation of all emergency department (ED) presentations to most public ED centres in NSW | ICD-10-AM, ICD-9, SNOMED CT-AU | Mental ill-health, neurodevelopmental comorbidities |
| The Medicare Benefits Schedule (MBS) | 1 Jan 2000-31 Dec 2018 | Information on provider, service and fees of all medical services subsidized by the Australian Government’s Medicare program | No diagnoses recorded | Mental ill-health |
| Disability Services Minimum Dataset (DS-MDS) | 1 Jul 2003-30 Jun 2016 | People receiving services from the NSW Department of Ageing, Disability and Home Care or a non-government organisation funded by them | - | Neurodevelopmental comorbidities |
| NSW Targeted Specialist Education Services (TSS) | 1 Jan 2007-23 Jul 2019 | Students with intellectual disability who received special education services from public primary and secondary schools | - | Neurodevelopmental comorbidities |
| NSW Corrective Services State-wide Disability Services (SDS) | 8 Jan 2001-1 Apr 2019 | Offenders who received disability services while in custody or in the community | - | Neurodevelopmental comorbidities |
| NSW Public Guardian | 1989-2016 | People aged over 16 who were appointed a Public Guardian due to lack of decision-making ability | - | Neurodevelopmental comorbidities |
| NSW Ombudsman | 5 Dec 2002-30 Dec 2018 | Information on deaths in residential care reviewed by the Ombudsman | - | Neurodevelopmental comorbidities |

Abbreviations: ICD-10-AM= International Classification of Diseases and Related Health Problems, 10th Revision, Australian Modification; ICD-9= International Classification of Diseases and Related Health Problems, 9th Revision; SNOMED CT-AU= Systematized Nomenclature of Medicine Clinical Terms Australian Extension
