# Supplementary material for: Predictors of mental illness onset in adolescents and adults with intellectual disability: A retrospective cohort study in New South Wales, Australia
Source: Aust N Z J Psychiatry. 2025 Sep 28;59(12):1095–105. doi: 10.1177/00048674251374483 (PMC12634903; doi:10.1177/00048674251374483)
Supplement: sj-docx-2-anp-10.1177_00048674251374483 – Supplemental material for Predictors of mental illness onset in adolescents and adults with intellectual disability: A retrospective cohort study in New South Wales, Australia [file sj-docx-2-anp-10.1177_00048674251374483.docx]

**Supplementary Material 2: Variable definitions**

**Outcomes**

Non-organic and non-neurodevelopmental psychiatric disorders are ascertained from the APDC, MHAMB, and EDDC datasets using the following diagnostic codes:

ICD-10-AM: F10-F63, F65-F69, F91-F94, F98.0-F98.3, F99, R45.81, X60-X84, Y87.0, U79.1-U79.3

ICD-9: 291-292, 295-298, 300-301, 302.0-302.4, 302.7, 302.81-302.84, 302.89, 302.9, 303-309, 311-313, 316, V62.84, E950-E959

SNOMED CT-AU codes from the EDDC were converted to ICD-10-AM as follows:

1. Export unique SNOMED codes from EDDC dataset.
2. Use SnoMAP Starter AU tool to map SNOMED codes to ICD-10-AM equivalents (Lawley et al., 2017).
3. Upload non-matched SNOMED codes to the Snapper: Map tool (Australian e-Health Research Centre, n.d.), and convert them to SNOMED-CT version 2020-03-31 (the version used in SnoMAP Starter).
4. Upload converted SNOMED codes to SnoMAP Starter and rerun mapping procedure, add newly mapped codes to original map.
5. Manually screen unmapped codes related to mental health and assign ICD-10-AM equivalents if applicable.

All MHAMB services and the following MBS services are included in the definition of “any mental illness”:

288, 289, 291, 293, 296, 297, 299, 300, 302, 304, 306, 308, 310, 312, 314, 316, 318, 319, 320, 322, 324, 326, 328, 330, 332, 334, 336, 338, 353, 355, 356, 357, 358, 359, 361, 364, 366, 367, 369, 370, 855, 857, 858, 861, 864, 866, 2574, 2575, 2577, 2578, 2700, 2701, 2702, 2704, 2705, 2707, 2708, 2710, 2712, 2713, 2715, 2717, 2719, 2721, 2723, 2725, 2727, 6018, 6019, 6023, 6024, 6025, 6026, 6029, 6031, 6032, 6034, 6035, 6037, 6038, 6042, 10956, 10968, 14224, 20104, 80000, 80001, 80005, 80010, 80011, 80015, 80020, 80021, 80100, 80101, 80105, 80105, 80110, 80111, 80115, 80115, 80120, 80121, 80125, 80126, 80130, 80135, 80136, 80140, 80145, 80146, 80150, 80151, 80155, 80160, 80161, 80165, 80170, 80171, 81325, 81355

**Predictors**

Neuropsychiatric comorbidities are ascertained from ICD-10-AM diagnostic codes in the APDC and MHAMB datasets, ICD-9 and SNOMED CT-AU (converted to ICD-10) codes in the EDDC dataset, and relevant variables from disability-specific datasets including the Disability Services Minimum Dataset (DS-MDS), NSW Targeted Specialist Education Services (TSS), NSW Corrective Services State-wide Disability Services (SDS), NSW Ombudsman, and NSW Public Guardian.

|  | ICD-10-AM | ICD-9 | Other datasets |
| --- | --- | --- | --- |
| Autism | F84 | 299 | DS-MDS: Primary disability type= “Autism”  TSS: Disability type= “Autism”  SDS: Autistic= “confirmed”  NSW Ombudsman: Manual coding of free text  NSW Public guardian: Manual coding of free text |
| ADHD and learning disorders | F80, F81, F90 | 314, 315 | DS-MDS: Primary disability type= “Specific learning/Attention Deficit Disorder”  TSS: Disability type= “Language”  SDS: Speech disability= “expressive”, “comprehension”  NSW Ombudsman: Manual coding of free text  NSW Public Guardian: Manual coding of free text |
| Cerebral palsy | G80, U80.4 | 343 | NSW Ombudsman: Manual coding of free text  NSW Public Guardian: Manual coding of free text |
| Epilepsy | G40, G41, U80.3 | 345 |  |
| Down syndrome | Q90, U88.2 | 758.0 | NSW Ombudsman: Manual coding of free text  NSW Public Guardian: Manual coding of free text |
| Other congenital syndromes^1^ | D82.1, P04.3, Q85.1, Q86.0, Q87.14, Q87.84, Q91, Q95.2 | 279.11, 758.1, 758.2, 759.5, 759.81, 759.83, 760.71 | NSW Ombudsman: Manual coding of free text  NSW Public Guardian: Manual coding of free text |

^1^ Includes fragile X syndrome, Prader-Willi syndrome, foetal alcohol spectrum disorder, tuberous sclerosis, William’s syndrome, DiGeorge syndrome (22q11.2 deletion syndrome), Edwards syndrome (trisomy 18), Patau syndrome (trisomy 13).

Physical comorbidities include the following long-term conditions, identified using ICD-10-AM diagnostic codes in the APDC. The start date of the first episode with an eligible diagnosis recorded is used as the date of onset. The number of physical comorbidities is calculated at 1 Jan of each year in the study period, as the total number of conditions that each individual has acquired from 1 Jul 2001 to 31 Dec of the previous year.

| Condition | ICD-10 codes |
| --- | --- |
| Anaemia | D50-61, D63, D64 |
| Coagulopathy | D65-68, D69.1, D69.3, D69.4, D69.5, D69.6, D69.8 |
| Protein-energy deficiency | E40-E46 |
| Nutritional deficiencies | E50-E64 |
| Thyroid disorders | D34, E00-E05, E06.2, E06.3, E06.5, E07, E35.0, E89.0 |
| Obesity | E66, U78.1 |
| Hyperlipidaemia | E78 |
| Hypertension | I10, I15, U82.3 |
| Hypotension | I95 |
| Non-metastatic cancer | C00-C76, C80-C97, D37-D48 |
| Metastatic cancer | C77-C79 |
| Coronary heart disease | I20-I25, Z95.1, Z95.5, U82.1 |
| Heart failure | I50, U82.2 |
| Rheumatic and valvular diseases | I05-I09, I34-I39, Q22, Q23, Z95.2, Z95.3, Z95.4 |
| Arrhythmia | I44, I45, I47-I49 |
| Inflammatory heart disease and cardiomyopathy | I31.0, I31.1, I31.8, I31.9, I42, I43 |
| Peripheral vascular disease | I70, I72-I74, I77.1, I79.1, I79.2, I79.8, I80-I82, I87.2, I99, Q27, Q28 |
| Pulmonary heart diseases | I26, I27 |
| Other cardiovascular diseases | I11, I13, I28, I51, I52, I71, I78.0, I98.0, I98.1, Q20, Q21, Q24-Q26 |
| Stroke/transient ischaemic attack | G45, G46, I60-I69 |
| Diabetes | E09-E14, O24 |
| Oral and upper digestive tract diseases | I85, I86.4, I98.2, I98.3, K04.5, K05.1, K05.3-K05.6, K20, K21, K22.0-K22.2, K22.4, K22.5, K22.7-K22.9, K23, K25.4-K25.9, K26.4-K26.9, K27.4-K27.9, K28.4-K28.9, K29.2-K29.9 |
| Inflammatory and other bowel diseases | K50-K52, K55.1, K55.8, K57, K58, K90, U84.1, U84.2 |
| Chronic liver disease | B18, K70, K71.1, K71.3, K71.4, K71.5, K71.7, K72.1, K72.9, K73, K74, K75.3, K75.4, K75.8, K75.9, K76, K77.8, Q44.6, Z94.4, U84.3 |
| Chronic pancreas and gallbladder disease | K80, K81.1, K86.0, K86.1, K86.8, Q44.0, Q44.1, Q44.2, Q44.3, Q44.4, Q44.5, Q45.0 |
| Chronic constipation | K56.0, K56.4, K56.7, K59.0, K63.1, K63.4, K63.8 |
| Hearing loss | H80, H81.0, H90, H91, H93.3, H93.8, Q16, Z45.3, Z46.1, Z96.2, Z97.4 |
| Vision impairment | H25, H26, H28.0, H28.1, H28.2, H40, H42, H54.0, H54.1, H54.2, H54.4, H54.5, H54.6, H54.9 |
| Chronic kidney disease | E09.2, E10.2, E11.2, E12.2, E13.2, E14.2, I12, I13, I82.3, N01-N08, N11-N16, N18, N25.0, N25.1, N39.1, N39.2, Q60, Q61, Q63.8, Z49, Z90.5, Z99.2, U87.1 |
| Chronic urinary tract disease | B90.1, N30.1, N30.2, N30.3, N30.4, N31, N32.0, N32.3, N32.8, N32.9, N33, N35, Q54, Q62.0, Q62.1, Q62.2, Q62.3, Q62.4, Q62.7, Q62.8, Q64.0, Q64.1, Q64.3, Q64.4, Q64.5, Q64.6, Q64.7, Q64.8, Q64.9, Z90.6 |
| Urinary incontinence | N39.3, N39.4, Z90.6 |
| Prostate conditions | D29.1, N40, N41.1, N41.8, N41.9 |
| Connective tissue disorders | L94, M02.3, M05, M06, M08, M09, M30-M36, M61, M65.2, M65.3, M65.4, M70.0, M72.0, M72.2, M72.4, M73, M75.0, M75.1, M75.2, M75.3, M75.4, M75.5, M75.6, M76, M77, U86.1, U86.3 |
| Other arthritis and related disorders | M07, M10-M19, U86.2 |
| Chronic pain | B02, G44, M25.5, M40, M41, M45-M54, M79.1, M79.2, M79.6, M79.7, M99, Q67.5 |
| Deformities of joints/limbs | M20, M21, Q65, Q66, Q68, Q71-Q74, Q77, Q78, Q79.6, Q79.8, Z89 |
| Osteoporosis | M80-M82, M85, U86.4 |
| Other musculoskeletal disorders | M22-M24, M25.2, M25.3, M84.1, M89, M91, M93, M94, M96 |
| Dementia | A81.0, F00-F03, F05.1, F06.7, G30, G31, U79.1 |
| Progressive neurological disorders | G10, G11.1-G11.9, G12, G20-23, G32, G35, G37, G71.0, U80.1, U80.2 |
| Migraine | G43 |
| Neuropathies | B91, G14, G50-G64 |
| Non-traumatic paralysis excluding stroke | G81-G83, U80.5 |
| Sleep disorders | G47 |
| Other neurological conditions | B90.0, D32, D33, G04.1, G09, G11.0, G13, G25.0, G25.2, G25.3, G25.5, G25.8, G25.9, G26, G70, G71.1, G71.2, G71.3, G71.8, G71.9, G72.3, G72.4, G72.8, G72.9, G90, G91, G93.1, G93.3, G95, G99, Q85 |
| Chronic gynaecological disorders | E28.2, N70.1, N71.1, N73.1, N73.4, N76.1, N76.3, N80 |
| Asthma | J45, U83.3 |
| Chronic pulmonary diseases | J40-J44, J47, J60-J67, J68.4, J70.1, J70.3, J84, J95.3, J96.1, J98.0, J98.2, J98.3, J98.4, J98.5, J98.6, J98.8, J98.9, Q33, Q34, Z90.2, U83.1, U83.2, U83.4, U83.5 |
| Upper respiratory diseases | J30-J32, J34.1, J34.2, J34.3, J35, J37, J38.0, J38.6, J95.5 |
| Chronic skin conditions | L20, L21, L26, L28-L30, L40, L41 |
| Multi-system disorders | B20-B24, D80-86, E84, O98.7, Q87, Z94 |

**References:**

Australian e-Health Research Centre (n.d.) *Snapper*. Available at: <https://ontoserver.csiro.au/site/our-solutions/snapper/> (accessed 8 November).

Lawley M, Truran D, Hansen D, et al. (2017) SnoMAP: Pioneering the Path for Clinical Coding to Improve Patient Care. *Integrating and Connecting Care*. DOI: 10.3233/978-1-61499-783-2-55. 55-62.
