# Supplementary material for: Predictors of mental illness onset in adolescents and adults with intellectual disability: A retrospective cohort study in New South Wales, Australia
Source: Aust N Z J Psychiatry. 2025 Sep 28;59(12):1095–105. doi: 10.1177/00048674251374483 (PMC12634903; doi:10.1177/00048674251374483)
Supplement: sj-docx-3-anp-10.1177_00048674251374483 – Supplemental material for Predictors of mental illness onset in adolescents and adults with intellectual disability: A retrospective cohort study in New South Wales, Australia [file sj-docx-3-anp-10.1177_00048674251374483.docx]

**Supplementary Material 3: Directed acyclic graphs and variables in individually adjusted models**

**Table S3.1**

*Included variables in individually adjusted models of any mental ill-health*

| Variable of interest | Included variables |
| --- | --- |
| Female sex | Sex |
| Remoteness (ref: major cities) | Sex, Indigenous, remoteness, IRSD |
| Inner regional |  |
| Outer regional/remote/very remote |  |
| IRSD (ref: first quintile) |  |
| Second quintile |  |
| Third quintile |  |
| Fourth quintile |  |
| Fifth quintile (least disadvantaged) |  |
| Neuropsychiatric comorbidities |  |
| Autism | Sex, autism, ADHD and learning disorders, cerebral palsy, epilepsy, Down syndrome, other congenital syndromes |
| ADHD and learning disorders |  |
| Cerebral palsy |  |
| Epilepsy |  |
| Down syndrome | Down syndrome |
| Other congenital syndromes | Sex, other congenital syndromes |
| Physical comorbidity (ref: none) | Sex, Indigenous, remoteness, IRSD, autism, cerebral palsy, epilepsy, Down syndrome, other congenital syndromes, physical comorbidity |
| 1 condition |  |
| 2 conditions |  |
| 3 or more conditions |  |

**Figure S3.1**

*Directed acyclic graph (DAG) of relationships between demographic variables and any mental ill-health*
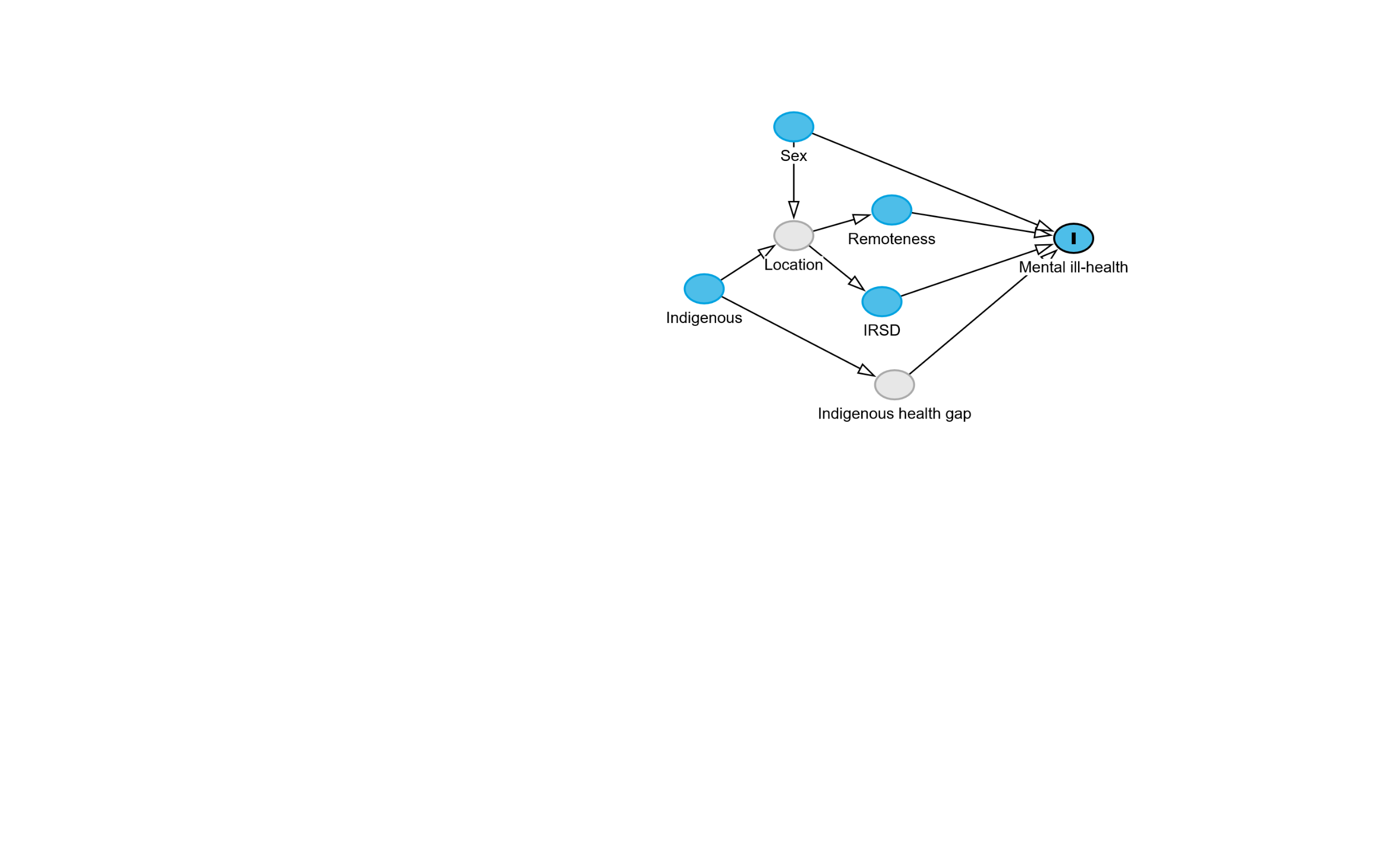


*Note.* Arrows indicate direction of causal relationship. Grey indicates unobserved variables used to demonstrate relationship between measured variables.

**Figure S3.2**

*Directed acyclic graph (DAG) of relationships between neuropsychiatric conditions and any mental ill-health*


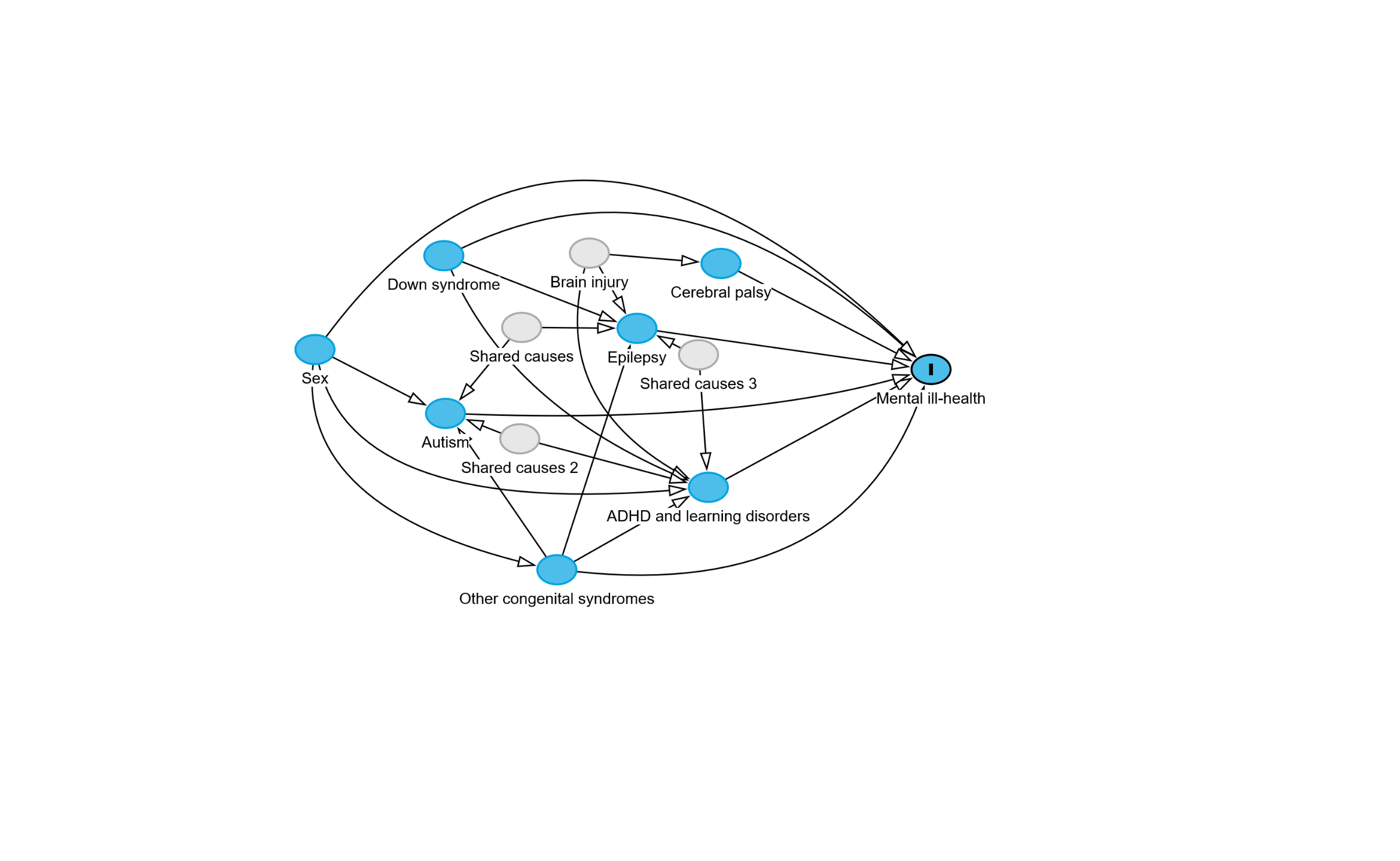


*Note.* Arrow indicates direction of causal relationship. Grey indicates unobserved variables used to demonstrate relationship between measured variables.

**Figure S3.3**

*Directed acyclic graph (DAG) of relationships between all variables and any mental ill-health*


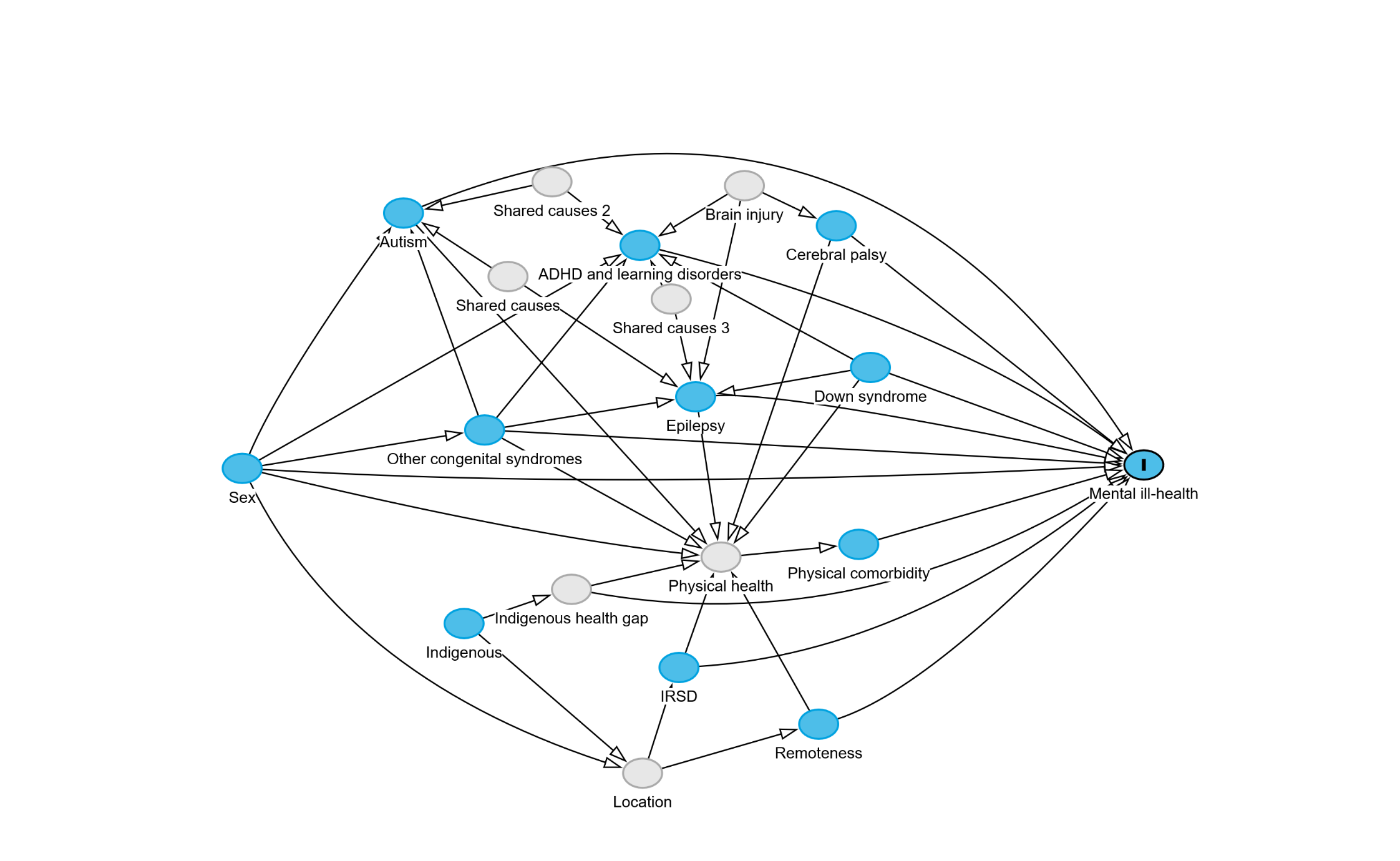


*Note.* Arrow indicates direction of causal relationship. Grey indicates unobserved variables used to demonstrate relationship between measured variables.

**Table S3.2**

*Included variables in individually adjusted models of serious mental illness*

| Variable of interest | Included variables |
| --- | --- |
| Female sex | Sex |
| Remoteness (ref: major cities) | Sex, Indigenous, remoteness, IRSD |
| Inner regional |  |
| Outer regional/remote/very remote |  |
| IRSD (ref: first quintile) |  |
| Second quintile |  |
| Third quintile |  |
| Fourth quintile |  |
| Fifth quintile (least disadvantaged) |  |
| Neuropsychiatric comorbidities |  |
| Autism | Sex, autism, ADHD and learning disorders, cerebral palsy, epilepsy, Down syndrome, other congenital syndromes |
| ADHD and learning disorders |  |
| Cerebral palsy |  |
| Epilepsy |  |
| Down syndrome | Down syndrome |
| Other congenital syndromes | Sex, other congenital syndromes |
| Physical comorbidity (ref: none) | Sex, Indigenous, remoteness, IRSD, autism, cerebral palsy, epilepsy, Down syndrome, physical comorbidity^1^ |
| 1 condition |  |
| 2 conditions |  |
| 3 or more conditions |  |
| Past mental ill-health | Sex, Indigenous, remoteness, IRSD, autism, ADHD and learning disorders, Down syndrome, physical comorbidity, past mental ill-health^2^ |

^1^Other congenital syndromes is omitted from this model due to insufficient variation resulting in failure to converge.
^2^Cerebral palsy, epilepsy, and other congenital syndromes are omitted from this model due to insufficient variation resulting in failure to converge.

**Figure S3.4**

*Directed acyclic graph (DAG) of relationships between all variables and serious mental illness*


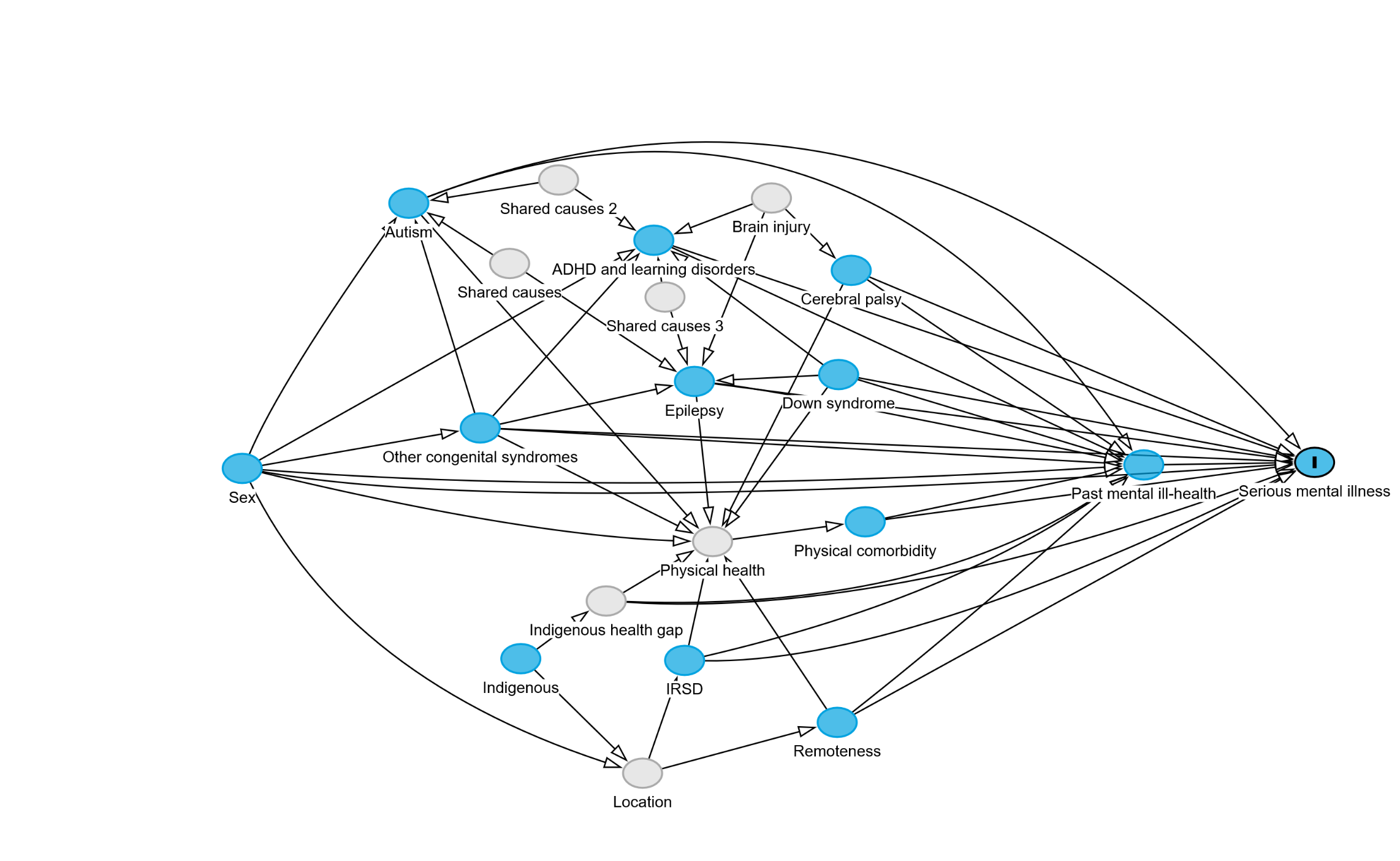


*Note.* Arrow indicates direction of causal relationship. Grey indicates unobserved variables used to demonstrate relationship between measured variables.
